# The role of the microbiome in gynecological cancers: implications for diagnosis and treatment

**DOI:** 10.3389/fimmu.2026.1718883

**Published:** 2026-04-23

**Authors:** Precious Adeoye Oyedokun, Bobola Timothy Oyeleke, Onigbinde Oluwanisola Akanji, Abraham Ololade Oyelaran, Kehinde Philip O, Grace Bosede Akanbi, Philip Olagbede Oyedokun, Marvelous Dasola Oyedokun, Chikezie Chinelo Naomi, Oyeniran Oluwatosin Imoleayo, Roland Eghoghosoa Akhigbe

**Affiliations:** 1 Department of Physiology, Ladoke Akintola University of Technology, Ogbomoso, Oyo State, Nigeria; 2 Reproductive Biology and Toxicology Research Laboratory, Oasis of Grace Hospital, Osogbo, Osun State, Nigeria; 3 Axon Plus Consulting, Ogbomoso, Oyo State, Nigeria; 4 Department of Medical Laboratory Science, Ladoke Akintola University of Technology, Ogbomoso, Oyo State, Nigeria; 5 Department of Anatomy, Nile University, Abuja, Nigeria; 6 Department of Computer Science, Ladoke Akintola University of Technology, Ogbomoso, Oyo State, Nigeria; 7 Department of Nursing Science, Ladoke Akintola University of Technology, Ogbomoso, Oyo State, Nigeria; 8 Department of Anatomy, University of Ilorin, Ogbomoso, Oyo State, Nigeria; 9 Department of Kinesiology and Integrative Physiology, Michigan Technological University, Houghton, MI, United States

**Keywords:** biomarkers, cancer, dysbiosis, microbial therapy, microbiota, probiotics

## Abstract

Gynecological malignancies such as cancer of the cervix, ovary, endometrium, vulva, and vagina pose a severe global health burden. Although conventionally attributed to genetic mutation, hormonal imbalance, and chronic viral infection, including high-risk human papillomavirus, recent evidence suggests that the human microbiome plays a central role in their pathogenesis and development. This review summarizes existing evidence that microbial dysbiosis, specifically the depletion of beneficial Lactobacillus species and overrepresentation of anaerobic organisms such as Fusobacterium, Atopobium, and Sneathia, is implicated in carcinogenesis pathways. These include chronic inflammation, immune modulation, loss of epithelial barrier integrity, microbial metabolite toxicity, and estrogen metabolism by the estrobolome. Dysbiosis in the gut and reproductive tract has been associated with HPV persistence, tumor microenvironment remodeling, and immune surveillance/therapy resistance. Consequently, microbial signatures are being investigated as a potentially successful non-invasive biomarker for early diagnosis, prognosis, and monitoring of therapy in gynecological oncology. In addition, emergent microbiome-based therapies are being considered as potential adjunct therapies, including probiotics, prebiotics, dietary manipulation, vaginal microbiota transplantation, and fecal microbiota transplantation. This review connects the basic research microbiome research to translational and clinical practice, identifies associated limitations, and highlights how it may transform gynecological cancer prevention, detection, and treatment.

## 1 Introduction

While cancer is a leading cause of death globally (1), gynecological cancers, including malignancies of the cervix, ovary, endometrium, vulva, and vagina, represent a significant public health burden and contribute substantially to cancer-related morbidity and mortality among women globally. Zhu et al. (2) reported that approximately 15.25 percent of new cancer cases in women were gynecological cancers in 2022. In addition, gynecological cancers are responsible for 15.77 per cent of cancer-related mortality, which is due to gynecological cancer pathology (2). According to GLOBOCAN 2020 data, cervical cancer alone accounts for over 600,000 new cases and 340,000 deaths annually, with the burden disproportionately affecting low- and middle-income countries (3). The global incidence of endometrial cancer in 2020 was estimated at 417,000 new cases, responsible for 97,000 deaths (4). Similarly, approximately 20,890 women will receive a new diagnosis of ovarian cancer (5).

While cervical cancer is primarily caused by persistent infection with high-risk human papillomavirus (HPV), other gynecological cancers such as endometrial and ovarian cancers are linked to hormonal imbalances, obesity, chronic inflammation, and hereditary mutations, notably in *BRCA1/2*, *PTEN*, and *MLH1* genes (6, 7). Historically, the etiological focus in gynecological oncology has centered on genetic and viral risk factors. However, more recently, the development of microbiome science has focused on non-genetic contributors to carcinogenesis, one of these being the human microbiome. The microbiome encompasses the natural and diverse community of microorganisms, bacteria, fungi, viruses, and archaea that live in mucosal and epithelial surfaces of the body, including the female reproductive tract. These microbial communities are involved in the regulation of immune responses, hormonal metabolism, epithelium barrier integrity, and local inflammatory responses (8, 9). Dysbiosis, a disturbance in the normal balance of microorganisms, has been implicated in a number of oncogenic processes, including chronic inflammation, alteration of cellular proliferation pathways and the suppression of anti-tumor immunity (10).

In cancers of gynecological interest, specific microbial profiles have been linked with development of the disease. For example, elevated amount of Lactobacillus species and reduced incidences of colonization from anaerobes such as Sneathia, Fusobacterium and Atopobium have been reported from cervical and endometrial cancer patients (11, 12). In the same vein, findings revealed there are differences between the composition of endometrial and ovarian microbiota of individuals with cancer and healthy subjects, suggesting microbial dysbiosis as a direct cause of carcinogenesis (13). Moreover, gut and the oral microbiomes are linked to hormone-related tumorigenesis via estrogen and systemic inflammation-dependent pathways (14, 15).

Despite the growing evidence of the involvement of the microbiome in gynecological cancers, its clinical implications are underexplored. This review addresses this gap by examining how microbial dysbiosis contributes to the development of cancers of the cervix, ovary, endometrium, vulva, and vagina. This review synthesizes literature to identify processes, immune disruption, hormonal imbalance, and chronic inflammation, and assesses the promise of microbial signatures as noninvasive biomarkers. This narrative review also discusses the relevance and effectiveness of emerging microbiome-based interventions, such as probiotics, prebiotics, and microbiota transplantation, which improve diagnosis, prognosis, and treatment outcomes, thereby filling the gap between research and clinical practice.

## 2 Background on the human microbiome

### 2.1 The lower reproductive tract microbiome

The usual healthy vaginal microbiome is mainly constituted of the representatives of the Firmicutes phylum, including *Lactobacillus crispatus, Lactobacillus gasseri, Lactobacillus iners, and Lactobacillus jensenii* (9, 16). The communities dominated by Lactobacillus have been reported to have a plethora of benefits that are manifested through the inhibition of inflammation and pathogen susceptibility and maintenance of a healthy microenvironment (17). The strains of Lactobacillus species can also vary in functional activity since some of them provide more benefits to the host, compared to others (18). *Lactobacillus crispatus* produces the most lactic acid of all vaginal Lactobacilli. These facilitates microbiome-mediated protection without activating inflammation to preserve vagina health (19). By contrast, communities of *Lactobacillus iners* are often transient and tend to be associated with anaerobic microbes such as *Gardnerella, Ureaplasma*, and *Prevotella* species, which have the capacity to generate bacterial vaginosis (18). Reduction in the abundance of *Lactobacilus* and increased population of the anaerobic taxa such as *Gardnerella*, *Sneathia*, *Prevotella*, and *Fusobacterium* induces disruption of the immune system and pro-inflammatory microenvironment that facilitates HPV acquisition, persistence, and impaired viral clearance; thus initiating cervical carcinogenesis (20).

### 2.2 Microbiota of the upper reproductive tract

Further, evidence reporting microbiota-driven carcinogenesis of the upper female reproductive tract (including the ovaries, fallopian tubes, and the uterus) abound overturning the traditional understanding that microbial colonization is restricted to the vagina (21–23). The endometrium may have taxa such as the *Fusobacterium nucleatum, Atopobium vaginae, Gardnerella* and *Porphyromonas* associated with hyperplasia and malignancy (24). Fallopian tube lesions, including the serous tubal intraepithelial carcinoma (STIC) may also present microbial signatures (25). Inflammatory or dysbiotic taxa have been found in ovarian tissue and ascites such as the *Proteobacteria, Rikenellaceae, Dialister, Akkermansia and Chlamydia trachomatis* (23).

## 3 Diagnostic roles of the microbiome in cancers of the cervix, ovary, endometrium, vulva, and vagina

Traditionally, bacteria was studied mainly with microscopes. However, after the emergence of modern sequencing technologies referred to as the next-generation sequencing (NGS), has improved the understanding of the human microbiome and its role in health and disease (26). Modern molecular techniques like 16S rRNA gene sequencing, whole-genome microbial sequencing, and metagenomic analyses have revolutionized the traditional microbiological work and revealed the complexity of microbial life, genetic and metabolic potentials (27). Polymerase chain reaction (PCR), DNA hybridization or fingerprinting has been used to characterize the microbe 16S ribosomal RNA (rRNA) (27).

Moreover, next generation sequencing has assisted in the unraveling of unique characterization of microbiotas in both benign and malignant gynecologic diseases, through the identification of new links between bacteria species and gynecologic cancer. The microbiome intersect could also be a significant way to enhance the outcomes of gynecologic cancer through early diagnosis, therapeutic intervention, and modulation (28, 29).

Distinct uterine signatures of microbes have also been detected in endometrial hyperplasia and endometrial carcinoma. Endometrial cancer has been linked to enrichment of *Fusobacterium nucleatum, Atopobium, Porphyromonas, and Prevotella* and higher microbial diversity compared to normal endometrium (28, 29). These microbial shifts are correlated with inflammatory pathways and altered immune signaling, implying the potential of diagnosis and differentiation between benign endometrium and premalignant and malignant endometrium. Thus, cervical or uterine sampling may present early detection strategy using minimally invasive approaches (16, 30).

Ascitic and tumor-resident microbiomes present emerging biomarkers for ovarian cancer although available literature report show that early diagnosis is difficult (31). Enrichment of *Proteobacteria, Rikenellaceae, Dialister* and *Akkermansia* has been reported in ovarian tumor tissue and ascites. Unique microbial fingerprints linked to high-grade serous carcinoma (28). Microbiota-associated inflammatory and metabolic factors in ascites have been associated with tumor aggressiveness and response to chemotherapy (29). These signatures may help distinguish between malignant and benign adnexal masses, to predict treatment outcomes.

Though there is paucity of evidence on the diagnostic role of the microbiome in vulvular cancer, some reports have established significant differences in the microbiome in vulvar cancer. According to Ayala and Fatehi (32) HPV-associated vulvar intraepithelial neoplasia (VIN) showed increased anaerobic dysbiosis (Gardnerella, Prevotella) and loss of protective commensals (28). Non-HPV vulvar squamous cell carcinoma due to chronic inflammatory dermatoses such as lichen sclerosus shows distinct changes in microbes, including changes in the composition of Staphylococcus (33). Indicating promising diagnostic application, these features may be helpful in differentiating VIN from benign vulvar dermatoses.

Vaginal cancer has similar etiologic and microbial overlaps to cervical cancer. It has been demonstrated that the vaginal microbiome contributes to cervical carcinogenesis significantly (34). The HPV 16 and HPV 18 and high-risk human papillomavirus have been labeled as the oncogenic initiators of cervical cancer (CC). Studies report loss of *Lactobacillus* dominance, dominance of high-risk HPVs associated anaerobic bacteria such as *Sneathia* and *Gardnerella* which are microbial patterns associated with HPV persistence and progression to vaginal intraepithelial neoplasia (35).

## 4 Microbiome dysbiosis and gynecological carcinogenesis

A mounting body of evidence has shown that the pro-tumorigenic environment arises from microbial alterations and attendant host cellular processes, driven by chronic inflammation, microbial toxin production, immune modulation, and hormonal dysregulation as mechanistic factors (36). Microbial dysbiosis is marked by a microbiome shift that involves the overriding of healthy, symbiotic bacteria (including *Lactobacillus* species) by increased anaerobic and pathogenic taxa such as Gardnerella, Prevotella, Sneathia, Fusobacterium, Atopobium, *Fusobacterium nucleatum*, *Peptostreptococcus*, *Mobiluncus*, and *Sneathia* spp (37). According to the findings of Suárez et al. (38) microbial dysbiosis diminishes the mucosal integrity and disrupts immune homeostasis by facilitating pathogen colonization and causing chronic inflammation.

The presence of the commensal anaerobe, *Fusobacterium nucleatum*, is a particularly striking indicator of OGR microbial interplays in gynecologic tumor tissues (39). Periodontal disease and colorectal cancer are both linked to this Gram-negative, anaerobe bacterium which is normally found in the oral cavity (40). The presence of mutations in gynecologic tumors suggests translocation or systemic dissemination and it can also impact responses to immunity and to cancer growth (38).

In addition to noticeable expression of pro-inflammatory and immune molecules associated with tumors, Castellarin et al. (41) showed F. nucleatum DNA and RNA in ovarian and endometrial cancer tissues. F. nucleatum adheres via FadA adhesin to bind to epithelial cells which initiate rapid cell growth and invasion into surrounding tissues (42). It also causes an increase in myeloid-derived suppressor cells (MDSCs) and decreases the effectiveness of cytotoxic T-cells thus facilitating cancer cells evading the immune system (42). The ability of this oral pathogen to impact the tumor microenvironment establishes the systemic involvement of this oral microbiota in cancer development in the female reproductive system.

The results show that adaptations of the microbiota in the gut may provoke alterations in the organs distant to the mouth such as the reproductive system. These alterations have the potential to deteriorate the tissues that line the reproductive tract, lead to chronic inflammation and genomic changes that eventually increase susceptibility to gynecologic cancer. Altogether, The mechanistic connection between microbial dysbiosis and gynecological relies on immune dysregulation, humoral inflammation, aberrant estrogen signaling and microbial metabolism (43).

### 4.1 Mechanisms of microbiome dysbiosis in gynecological carcinogenesis

#### 4.1.1 Microbiota-derived metabolite-induced carcinogenesis

The principal mechanism involves the translocation and systemic circulation of metabolites produced by microbes, especially short-chain fatty acids (SCFAs), including butyrate and propionate, secondary bile acids, lipopolysaccharides (LPS), and microbial nucleic acids (44). Due to the situation of microbial dysbiosis and compromised mucosal barrier integrity, these metabolites may penetrate into the blood circulation and have biological activity in other organs, e.g., in the female reproductive tract (45).

The LPS, a major constituent of Gram-negative bacterial outer membrane, has been widely known to induce tumorigenesis among these metabolites (46). Under physiological conditions, the intestinal epithelial barrier restricts the systemic dissemination of LPS (46). However, factors such as diet-induced dysbiosis, inflammation, antibiotic exposure, or stress can disrupt gut homeostasis, resulting in increased epithelial permeability (“leaky gut”) and enhanced translocation of LPS into the systemic circulation (47).

Once in circulation, LPS activates the immune system, more specifically, TLR4 of epithelial and immune cells within the female reproductive system. The activation of TLR4 signaling pathway results in the activation of NF-kB signaling pathway, which leads to the transcription of pro-inflammatory cytokines such as interleukin-6 (IL-6) and tumor necrosis factor alpha. These cytokines contribute to pro-tumorigenic microenvironment via the induction of chronic low-grade inflammation, DNA damage, enhanced cellular proliferation, angiogenesis and immune evasion, all of which are hallmarks of cancer progression (48).

SCFAs such as acetate, butyrate and propionate, essentially produced by microbial fermentation of dietary fibers, have a dual role depending on the context of microbiota balance and host response (49–51). Under eubiotic conditions, butyrate possesses anti-inflammatory and epithelial-protective activities through the regulation of the functions of T-regulatory (Treg) cells and integrity of the intestinal barrier (52).

**Figure 1:**
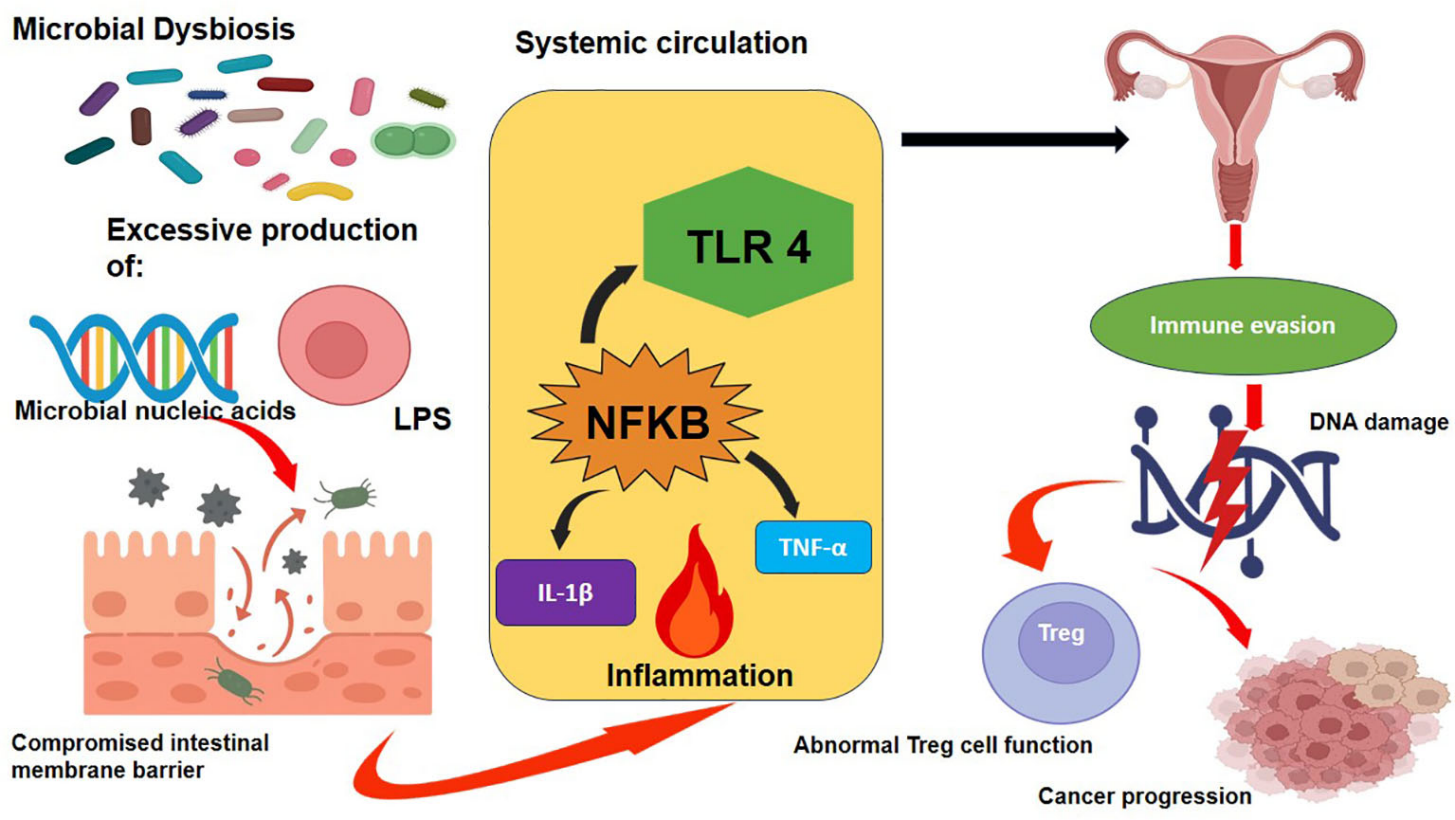
Mechanistic illustration of microbiota-induced metabolite carcinogenesis.

However, during dysbiosis, the relative production and signaling pathways of SCFAs may shift, resulting in immune dysregulation and facilitation of a tumor-permissive immune landscape (47) (**Figure 1**).

Gut Dysbiosis provokes excessive production of lipopolysaccharides (LPS), short-chain fatty acids (SCFA), microbial nucleic acids, and propionate. The dysfunctional intestinal barrier (leaky gut) allows the shuttle of these metabolites through systemic circulation to the reproductive system. LPS then activates Toll-like receptor 4 (TLR4) on the epithelial and immune cells inducing NF- kB signaling and pro-inflammatory cytokine IL-6 and TNF-a release. This promotes a tumor-supportive environment, chronic inflammation, DNA damage, immune evasion, and uncontrolled cell growth, contributing to carcinogenesis in the cervix, ovary, and endometrium.

#### 4.1.2 Chronic inflammation and immune dysregulation

**Figure 2:**
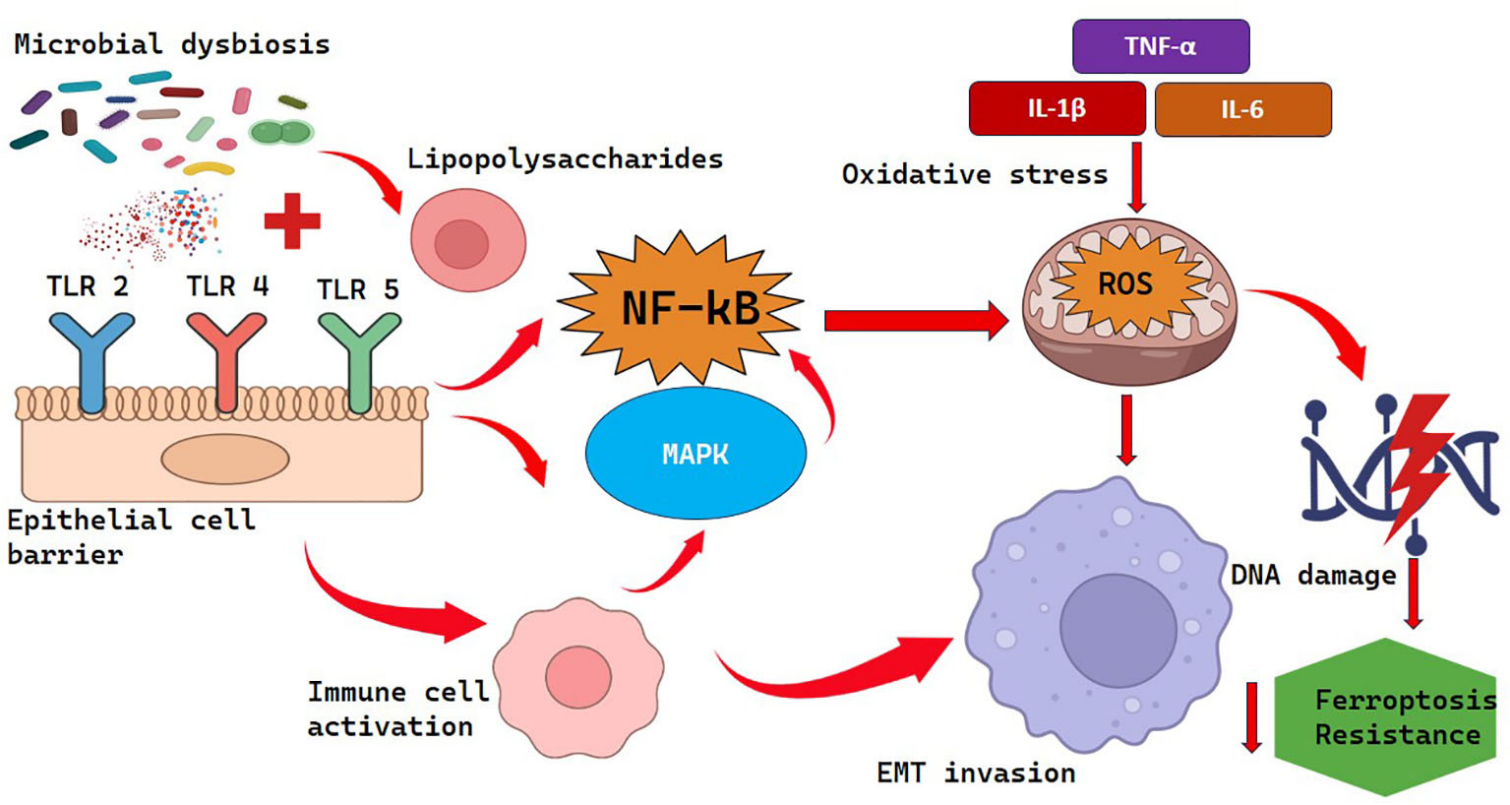
Microbiome-mediated inflammation and immune dysregulation.

Chronic inflammation is a central mechanism through which dysbiosis promotes carcinogenesis (**Figure 2**). Microbial-associated molecular patterns (MAMPs) including lipopolysaccharides (LPS) of Gram-negative bacteria, released by dysbiotic bacteria, stimulate pattern recognition receptors (PRRs) such as Toll-like receptors (TLRs) 2, 4, and 5 on epithelial and immune cells (53). This stimulation in turn initiates the signaling cascade within a cell, specifically, nuclear factor kappa-light-chain-enhancer of activated B cells (NF-kB) and mitogen-activated protein kinase (MAPK), which results in the production of pro-inflammatory cytokines (IL-6, IL-8, TNF-α) and chemokines (54–56).

According to Lu et al. (56), in endometrial cancer, a higher uterus microbial diversity was associated with the higher expression of IL-6, IL-8, and IL-17, which was positively related to the abundance of Micrococcus. This inflammatory condition elevates oxidative stress and produces reactive oxygen and nitrogen species (ROS/RNS) that damage DNA, induce genomic instability and mutations promoting tumor development and progression (57, 58).

It was found that the TLR4/NF-kB signaling stimulated by microbes showed an increased epithelial-mesenchymal transition (EMT), invasion, and metastasis in ovarian cancer (59, 60). In addition, Deng et al. (61) have found microbiota-derived metabolites in ascitic fluid that contribute to chronic inflammation and the resistance of tumor cells to a form of regulated cell death, ferroptosis, and thus to peritoneal dissemination.

The dysbiotic Gram-negative bacteria produce lipopolysaccharides (LPS), which engage in activating the pattern recognition receptors (TLRs 2,4 and 5) present on the epithelial and immune cells. This causes intracellular signal transduction, mainly involving NFKB and MAPK, which results in the secretion of pro-inflammatory cytokines, including IL-6, IL-8, and TNF-α. Chronic inflammation leads to the production of oxidative stress via reactive oxygen and nitrogen species (ROS/RNS), resulting in DNA dysfunction and genome instability (62). The alterations promote EMT invasion, impair resistance to ferroptosis and induce tumor progression and spread.

#### 4.1.3 Microbial toxins and oncogenic signaling pathways

**Figure 3:**
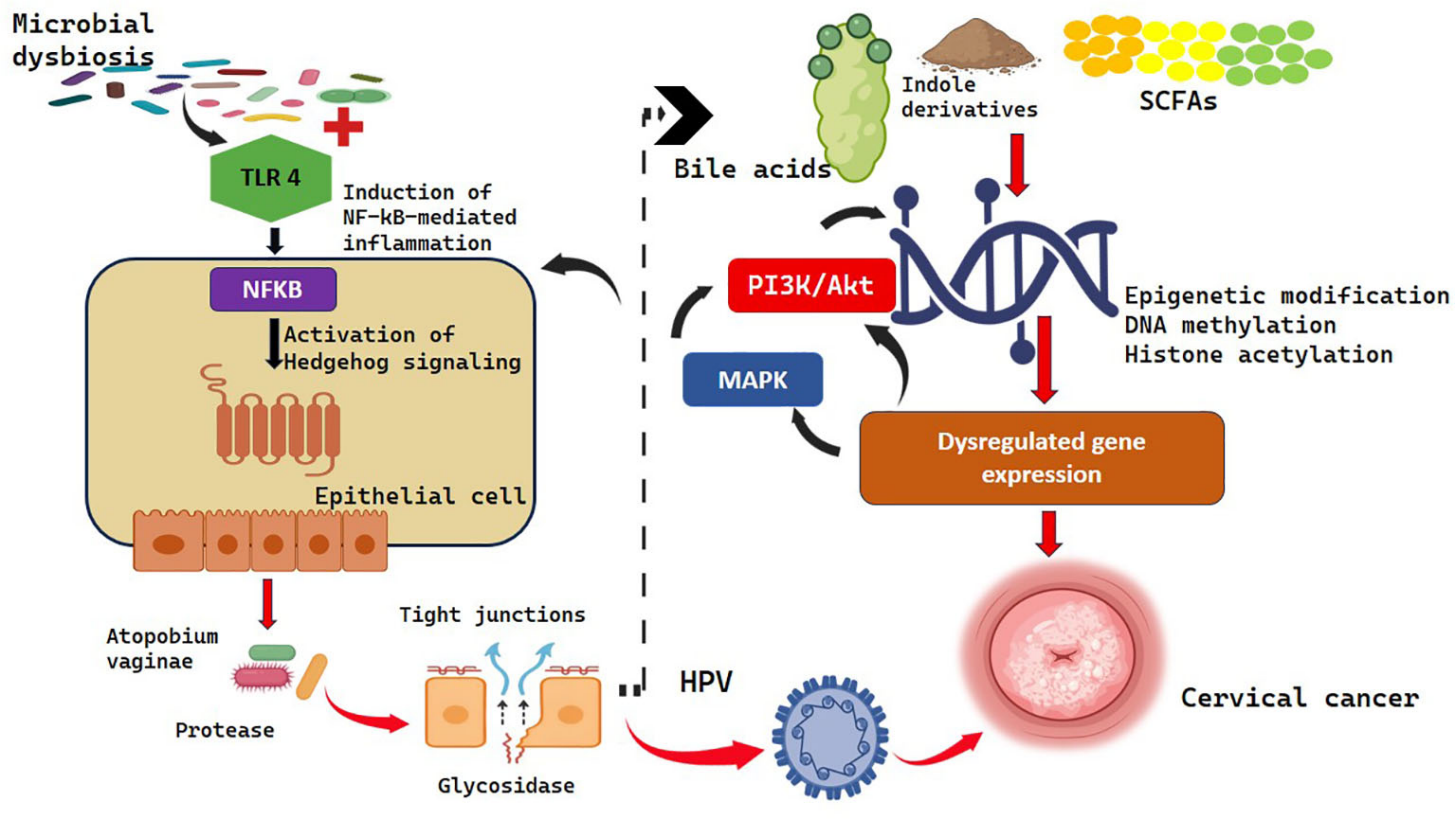
Microbial toxins and metabolite in oncogenic signaling.

The effects of microbial toxins and metabolites on oncogenic signaling pathways have profound impacts that directly affect the tumorigenic processes through inflammation-independent ways (**Figure 3**). The results of Hu et al. (63) revealed that the gut microbiome related to epithelial ovarian carcinoma in women activates the Hedgehog (Hh) signaling cascade through the interaction of TLR4 and the activation of nuclear factor kappa B (NF-kB) pathway. Such an interaction of molecules leads to increased proliferation of neoplastic cells, survival and metastatic potential in the tumor and, accordingly, tumor growth and spreading (47).

Also, secondary bile acids, indole derivatives, and short-chain fatty acids (SCFAs) are some microbial-derived metabolites that have been depicted to regulate DNA methylation and histone acetylation (64). Such epigenetic changes affect the patterns of gene expression that controls cellular proliferation, apoptosis, and immune-evasion processes (65; 66). Consequently, these metabolites lead to the development of an immunosuppressive tumor microenvironment and the oncogenic transformation (47).

The production of proteolytic enzymes and glycosidases by anaerobic bacteria like Atopobium vaginae weakens the integrity of cervical epithelial barrier by degrading the tight junction proteins and mucosal glycoproteins in cervical carcinogenesis (67, 68). Such an imbalance promotes the entry and maintenance of high-risk human papillomavirus (HPV) into the basal epithelial cells (69). Besides, A. vaginae causes the expression of aberrant mucin genes, which changes the composition of cervical mucus and facilitates the microenvironment favorable to viral persistence and oncogene expression (70; 53).

Oncogenic activity of HPV is enhanced by a dysbiotic microbial environment by stimulating intracellular signaling cascades PI3K/Akt and MAPK which increases cellular proliferation and suppresses programmed cell death (71; 72). This intermolecular communication is a factor of genomic unstability and malignant transformation of cervical cells (73). Therefore, microbial toxins and metabolites are key regulators of cervical carcinogenesis through their ability to mediate epithelial barrier destruction, enable viral oncogene expression and the ability to modulate host cell signaling, thereby enabling tumor initiation and progression.

The microbia toxins stimulate TLR4, initiating NF-kappa B and Hedgehog (Hh) signaling, which in turn promote tumor cell growth, survival and metastasis. Microbial metabolites Short chain fatty acids, bile acids, and indole metabolites that result in epigenetic modifications via DNA methylation and histone acetylation leading to aberrant gene expression, increased growth and immune response. Enzymes from anaerobic bacteria degrade epithelial barriers, allowing HPV entry and persistence. Dysbiosis further activates PI3K/Akt and MAPK pathways, driving genomic instability and malignant transformation and ultimately cervical cancer.

#### 4.1.4 Hormonal modulation via the estrogen-microbiota axis

**Figure 4:**
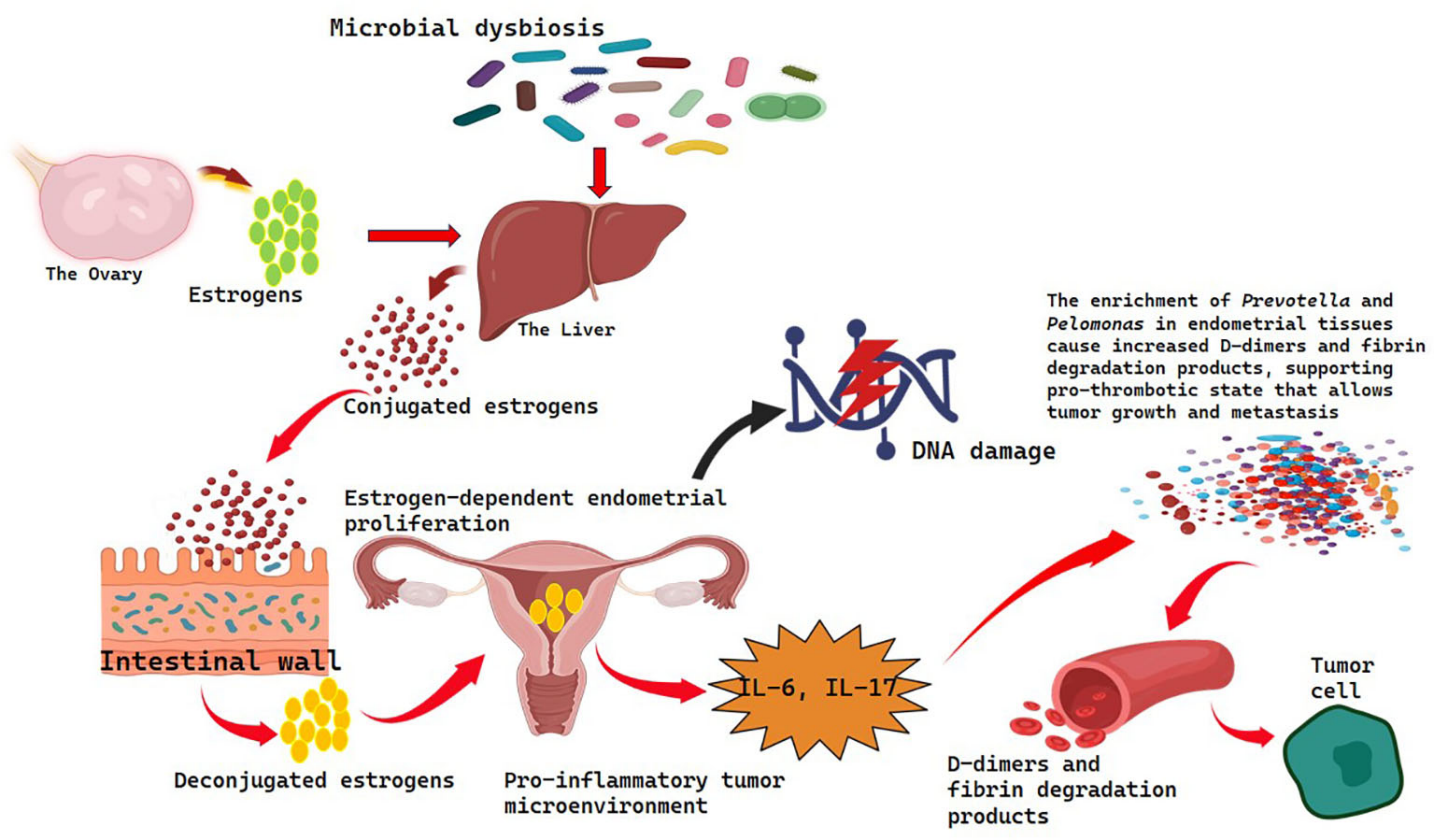
Hormone modulation via Estrogen-microbiota axis in gynecological cancers.

The estrobolome represents the collection of gut microbiota influences the systemic metabolism of estrogen; a functional ensemble of bacterial genes encoding estrogen-deconjugating enzymes specifically β-glucuronidase, which deconjugate estrogens within the intestinal lumen (74) **Figure 4**. The activity of this enzyme regulates the reabsorption of free estrogens and controls the amount of the circulating hormone in the body involved in physiological and pathological processes (75). Dysbiosis alters the intestinal microbiome altering the abundance and variety of bacteria producing the beta-glucuronidase activity which can be either stimulus or inhibitor of the reactivation of estrogen. According to the findings of Kwa et al. (75), the decreased microbial diversity, and significant decrease in the number of these prolific bacteria, is associated with low estrogen reactivation leading to the low estrogen levels in the body. On the other hand, some dysbiotic conditions can increase estrogen deconjugation and increase circulating estrogens (76).

This dysfunction estrogen metabolism has far-reaching consequences on estrogen dependent gynecological cancers such as endometrial cancer and ovarian cancer. Increased systemic concentration of estrogen stimulates endometrial proliferation and can induce carcinogenesis, as the proliferation and inhibition of apoptosis and DNA damage in the hormonally responsive tissues (77).

The gut and vaginal microbiota are connected through systemic immune pathways that influence the immune behavior following disturbance at the other site (78). Gut dysbiosis is conducive to translocation of microbial products such as lipopolysaccharides, peptidoglycan, and short-chain fatty acids into the circulation, and they modulate the cytokine profile, activation of dendritic cells, and polarization of T cells (79). These systemic immune changes may modify mucosal immunity in the cervicovaginal tract, including the integrity of the epithelium, leading to the recruitment of innate immune cells, and antiviral responses that play an important role in the control of HPV infection (78). Through immune dysregulation, the gut indirectly microenvironment may influence vagina carcinogenesis may be more permissive to inflammation, oncogenic virus persistence, and tumor-promoting pathways (80).

Microbial dysbiosis not only alters hormonal regulation but also inflammatory and coagulation cascades that further worsen the development of tumors in endometrial cancer. Lu et al. (56) revealed that endometrial cancer patients experienced a greater microbial diversity in the uterus than benign cases and the upregulation of pro-inflammatory cytokines interleukin-6 (IL-6) and interleukin-17 (IL-17).

Enrichment of other genera such as Prevotella and Pelomonas in tissues of the endometrial cancer was reported in Leoni et al. (81). The enrichment of Prevotella was also positively correlated with serum indicators of coagulopathy, namely, D-dimer and fibrin degradation products, which indicates an interrelation between tumor-related coagulopathy and microbial dysbiosis. This pro-thrombotic state may promote tumor growth and metastasis by facilitating angiogenesis and protecting circulating tumor cells.

The activity of the gut microbiota in regulating the levels of estrogen is caused by β-glucuronidase. Dysbiosis can cause a decrease or an increase of systemic estrogen that affects the risk of cancer. Abnormal levels of estrogens accelerate the endometrial cell growth and impairment of DNA. Due to the presence of dysbiosis, increased levels and secretion of inflammation (IL-6, IL-17) and coagulopathy (e.g., via Prevotella) are also seen, resulting in a tumor-supportive, pro-thrombotic microenvironment that favors cancer progression.

### 4.2 Cancer-specific mechanistic insights

#### 4.2.1 Cervical cancer: microbiota and HPV persistence

Persistent infection with high-risk HPV types (such as HPV 16 and 18) is necessary however insufficient for cervical cancer development (82). Microbiome alterations critically influence HPV persistence and oncogenic progression by modulating local immunity and epithelial barrier function (83). Liang et al. (84) demonstrated that vaginal microbiota characterized by decreased Lactobacillus and increased anaerobic bacteria such as Gardnerella, Prevotella, and Sneathia were associated with HPV persistence following cervical conization. This dysbiosis elevates local pro-inflammatory cytokines (IL-6, TNF-α) and growth factors (VEGF, hepatocyte growth factor), which promote epithelial proliferation, angiogenesis, and inhibit apoptosis (85, 86). Luo et al. (87) further revealed that cervical cancer lesions contain hypoxic microniches that are enriched with dysbiotic microbiota which have the ability to suppress the activation of interferon beta (STING) pathway and upregulate programmed death ligand 1 (PD-L1) to enable immune evasion and tumor progression. Anaerobic bacterial metabolites break down epithelial links to allow HPV access to basal keratinocytes where integration of the virus and expression of the viral oncogenes leads to carcinogenesis (88).

#### 4.2.2 Ovarian cancer: microbial components in ascitic fluid and peritoneal spread

Ovarian cancer progression involves a distinct tumor-associated microbiome (oncobiosis) within tumor tissue, ascitic fluid, and peritoneal surfaces. Asangba et al. (89) identified enrichment of Proteobacteria, Rikenellaceae, Akkermansia, and pro-inflammatory taxa such as Dialister and Prevotella in ovarian cancer patients. These microbes activate TLR/NF-κB signaling, promoting EMT, invasion, and metastasis. Setayeshpour et al. (2025) characterized microbiota-derived metabolites in ascitic fluid that protect cancer cells from ferroptosis, enhancing peritoneal dissemination. The ascitic fluid microenvironment, enriched with microbial components, sustains chronic inflammation and immune modulation, favoring tumor survival and spread (90).

Antibiotic treatment targeting Gram-positive bacteria during chemotherapy led to decreased cancer progression and improved overall survival, underscoring the critical role of the microbiome in modulating treatment response and tumor biology (91).

Ovarian cancer development is associated with a unique tumor-associated microbiome (oncobiosis) in tumor tissue, ascitic fluid and peritoneum. 89 reported that ovarian cancer patients showed enrichment of Proteobacteria, Rikenellaceae, Akkermansia, and pro-inflammatory microbes taxa Dialister and Prevotella. These microbes trigger TLR/NF-kB signaling which induces EMT, invasion, and metastasis. Setayeshpour et al. (2025) Antibiotic treatment targeting Gram-positive bacteria during chemotherapy led to decreased cancer progression and improved overall survival, underscoring the critical role of the microbiome in modulating treatment response and tumor biology. The microenvironment of ascitic fluid containing an efficient collection of microbial components promotes chronic inflammation and immune regulation in favor of tumor survival and dissemination (Setayeshpour et al, 2025). Antibiotic intervention against gram-positive bacteria during the course of chemotherapy resulted decreased the progression of cancer and improved overall survival; thus establishing the importance of the microbiome in modulating antibiotic response, and tumor biology (91).

#### 4.2.3 Microbial dysbiosis and endometrial cancer

The gut microbiota has mechanistic impacts on endometrial cancer development which is dominated by their modulation of systemic estrogen level and inflammatory signaling. One important pathway is through the estrobolome-estrogen metabolites that are deconjugated by the b-glucuronidase family in the gastrointestinal tract (92). This enzymatic action promotes the enterohepatic recirculation of estrogens for maintenance of bioactive systemic estrogen status. Changes in the microbe populations, especially the decreased presence of bacterial species that produce beta-glucuronidase (Betadinae and Clostridium spp) leads to less estrogen reactivation and subsequent hormone levels in the blood (93). In an estrogen-responsive tissue such as the endometrium, where chronic exposure to estrogen is a known risk factor for carcinogenesis, the dysregulation of estrogen metabolism may have a pathophysiological impact. Beyond estrogen metabolism, the dysbiosis of gut microbes is responsible for low-grade systemic inflammation which accounts for low-grade systemic inflammation to tumorigenesis. Kuźmycz et al. (94) showed that endometrial cancer patients alteration in microbial diversity in the uterus with abundance of inflammatory taxa such as Micrococcus. This was accompanied by increased local expression of pro-inflammatory cytokines IL-6 and IL-17 which are linked to inducing oncogenic signaling pathways such as STAT3 and NF-kB that contribute to the proliferation, vasculogenesis and immune escape of tumors.

In parallel, Prevotella and Pelomonas were recently found by Li et al. (88) to be significantly enriched in the gut and uterine microbiome, respectively, of endometrial cancer patients. Prevotella abundance was positively correlated with systemic coagulation markers including D-dimer and fibrinogen, linking microbial composition to the prothrombotic state commonly observed in malignancy (88). Prevotella species are also associated with mucosal barrier disruption and endotoxin release, which may exacerbate systemic inflammatory responses via Toll-like receptor (TLR) activation and downstream cytokine cascades (95).

These mechanistic pathways suggest that microbial-derived modulation of estrogen bioavailability, inflammatory cytokine production, and pro-coagulant activity collectively contribute to a microenvironment conducive to endometrial carcinogenesis.

## 5 Diagnostic and prognostic implications

The escalating global burden of gynecological malignancies have generated increasing attention to identifying microbial biomarkers for the detection of gynecological cancers at an early stage. Cervical, endometrial and ovarian cancers contribute to high cancer-related morbidity and mortality rates globally, with cervical cancer being the fourth most prevalent cancer in women and ovarian cancer still being the most fatal gynecological malignancy (2, 4, 5). In cervical cancer, a significant body of evidence has indicated that the changes in the vaginal microbiota are closely related to carcinogenesis. Lactobacillus species, especially Lactobacillus crispatus, depletion and overgrowth of anaerobic bacteria, such as Gardnerella vaginalis, Atopobium vaginae, Sneathia sanguinegens, Fusobacterium nucleatum, and Prevotella species, are commonly found in women with high-grade cervical intraepithelial neoplasia and invasive cervical cancer (11, 34). Such microbial changes are not just spectators, but are thought to play an active role in cervical carcinogenesis by mechanisms that include chronic mucosal inflammation, epithelial barrier dysfunction, and inhibition of immune-mediated viral clearance. Notably, the alteration of microbiota may precede the occurrence of histological abnormalities, which is why it can be considered a potential biomarker of cervical cancer at an early stage (96, 97).

Microbial dysbiosis has also been associated with endometrial cancer beyond the cervix. Researchers have shown that there are differences in microbial signatures in the uterine microbiome of women with endometrial hyperplasia and endometrial carcinoma. According to Ying et al. (98), women with endometrial hyperplasia had altered microbial profiles with reduced microbial diversity and enrichment of pro-inflammatory bacteria. In the same vein, Lu et al. (56) found that the endometrial microbiome of women with endometrial cancer had high levels of Fusobacterium nucleatum and other anaerobes. Riganelli et al. (99) also supported these results by noting structural differences in the vaginal and endometrial microbiome that could influence fertility, and potentially the risk of endometrial neoplasia. In combination, these studies indicate that vaginal and endometrial niche-specific bacterial taxa may be used as non-invasive biomarkers of early cancer detection. As high-throughput sequencing improves, these microbial biomarkers can be used in the near future, and even replace current cytology and molecular diagnostic methods (16).

Microbiota-based risk stratification is another equally significant direction of individual cancer risk evaluation. Increasing evidence has demonstrated that the composition of vaginal microbiota can stratify women by their risk of cervical cancer development. Women with higher vaginal microbiome of Gardnerella, Sneathia, Prevotella, and Atopobium species have an increased likelihood of having persistent high-risk HPV infection and development of cervical intraepithelial neoplasia (16, 84, 100). Such microbiota patterns are reported to enhance pro-inflammatory milieu, weaken epithelial barriers, and disrupt local immune system, which contribute to viral persistence and neoplastic development. Conversely, HPV clearance and reduced neoplasia are linked with Lactobacillus-dominant microbiota, especially those with high L. crispatus abundance.

The microbiome risk stratification is not limited to cervical cancer. In endometrial and ovarian cancers, microbial communities can alter systemic hormonal pathways and immune responses, which are the key factors in cancer development. Ge et al. (77) emphasized the importance of gut microbiota in controlling estrogen metabolism via the estrobolome, which affects endometrial carcinogene. In addition, the findings of Alizadehmohajer et al. (96) suggested the potential of microbiota-based risk models in which the signature microbial data are combined with clinical data to increase cancer risk prediction and inform personalized preventive interventions.

Vaginal microbiome profiling has emerged as an effective non-invasive screening technique of gynecological cancer. The vaginal microbiota is in direct contact with the cervical and vaginal epithelial tissue that provides an easily accessible but highly informative perspective on the local disease processes. Han et al. (101) established that certain vaginal microbiome signatures can accurately differentiate healthy women, HPV-positive women, and women with advanced cervical lesions or invasive cancer. In their analysis, they demonstrated that Lactobacillus-depleted, anaerobe-enriched microbiota profiles, especially the Gardnerella, Sneathia, and Fusobacterium dominated ones were closely associated with cervical neoplasia irrespective of HPV status (34, 100).

The advantages of vaginal microbiome screening have been reported. Vaginal self-sampling is non-invasive, can be replicated easily, and can be used in large-scale screening, especially in underserved populations where screening using cytology is still minimal (2). Moreover, microbiome profiling offers more biological information than HPV detection, as it records microbial changes associated with inflammation and identifies host-microbiome interactions that promote carcinogenesis (97, 99). Such peculiarities make vaginal microbiome profiling a highly promising addition to the current screening methods used to detect early-stage cancers, and in HPV-negative patients.

The prognostic implication of microbiome signatures in treatment response, especially in ovarian and cervical cancer, has also generated consistent attention. Asangba et al. (89) demonstrated that specific microbial communities in the tissue of ovarian cancer and ascitic fluid were associated with low clinical outcomes and chemoresistance. Immune evasion, ferroptosis resistance, and activation of the epithelial-mesenchymal transition pathway were linked to the enrichment of *Fusobacterium nucleatum* and *Dialister* species and resulted in treatment failure. Similarly, Setayeshpour et al. (90) also concluded that microbial metabolites can protect ovarian cancer cells against ferroptosis and raise their resistance to chemotherapy, which proves the mechanistic importance of tumor-associated microbiota in controlling therapy responses.

Vaginal microbiota composition has been associated with therapeutic outcome, particularly in the elimination of HPV infection and the de-regression of lesions in cervical cancer. Lactobacillus-dominant microbiota is associated with high HPV clearance and positive outcomes to conservative therapy of high-grade cervical intraepithelial neoplasia in women (102, 103). Conversely, persistent HPV infection and increased treatment resistance are associated with dysbiotic vaginal microbiota, which is anaerobic overgrowth. These results indicate that microbiome profiling may be used as a predictive biomarker in clinical decision-making, allowing to select the best treatment options and determine patients who might need more aggressive or alternative treatment.

Lastly, though Lactobacillus depletion is a consistent marker of cervical carcinogenesis, this is not the same for other gynecologic cancers. In cervical cancer, the loss of Lactobacillus dominance is strongly associated with failure of HPV clearance, local inflammation, and the progression to high-grade lesions (104). On the other hand, laboratory investigations have shown variable microbial signature in ovarian cancer characterized by communities predominated by Proteobacteria or mixed anaerobic taxa rather than Lactobacillus species (105). Some studies report no significant depletion of Lactobacillus in ovarian tumor environments (106, 107). This discrepancy highlights that the microbiome changes are specific to cancer sites, influenced by the variations in microenvironments of tumors, anatomical proximity to the vagina, and sampling strategies. Therefore, caution is necessary in elaborating general conclusions about gynecological cancers.

## 6 Microbiome-targeted therapies

The mounting body of available research evidence has revealed the introduction of microbiome-targeted therapies in modulating microbiome-associated cancer risk (**Table 1**). Jiang *et* al. (108) reported the promising potential of probiotics, prebiotics, and synbiotics as emerging as microbiome-targeted therapies for modulating cancer risk through gut microbiota regulation, immune modulation, and metabolic interactions. These are novel biotechnological strategies that involves the administration of beneficial, live microbes (probiotics) with desired capability in improving gynecological cancers or non-digestible ingredients (prebiotics) demonstrated to stimulate the growth the development and activity of beneficial microbes (108). Alternatively, therapeutic interventions may be a combination of probiotics and prebiotics regarded as synbiotics (109). Their roles span prevention, adjuvant therapy, and mitigation of treatment side effects across multiple cancer types, supported by preclinical and clinical evidence (109).

**Table 1:** Probiotic interventions in microbiome-associated gynecological cancers.

Probiotic strain	Exposure (Route/dose)	Effect	Mechanism	Reference
*Lactobacillus casei* Shirota	Oral	Improved clearance of low-grade cervical lesions	Immunomodulation; enhanced HPV clearance	Verhoeven et al. (102)
*L. crispatus* CHEN-01	Vaginal transplantation	Decreased HPV load, reduced vaginal inflammation	Restoration of vaginal microbiota; anti-inflammatory activity	Liu et al. (103)
*L. gasseri* LGV03	Cervico-vaginal isolate	Inhibited cervical cancer cell growth	Modulation of epithelial innate immune responses	Gao et al. (112)
*L. rhamnosus* BMX-54	Vaginal (chronic vs. short-term)	Improved resolution of HPV-related cytological abnormalities	Long-term microbiome stability and host immunity modulation	Palma et al. (113)
*L. rhamnosus* BPL005	Vaginal	Protective against endometrial infections	Lowers vaginal pH, organic acid production, suppression of pathogens	Chenoll et al. (114)
*L. jensenii* culture supernatant	*In vitro*	Inhibited cervical cancer cell viability	Induced apoptosis; downregulated MMP9 and HPV oncogenes	Fan et al. (115)
*Lactococcus lactis* (vaginal isolate)	*In vitro*	Anti-ovarian cancer effects	Downregulated TLR-4, miR-21, miR-200b; induced apoptosis	Saadat et al. (116)
Recombinant *L. lactis* expressing HPV-16 E7	Nasal	Induced immunity against HPV-associated cervical cancer	Cellular and humoral immune responses	Li et al. (117)
*L. casei*, *L. paracasei* (milk-isolated)	*In vitro* (HeLa)	Inhibited cervical cancer cell proliferation	Induced apoptosis via pro-apoptotic gene expression	Wang et al. (118)Bi et al. (119)
*L. delbrueckii* subsp. *lactis*	Oral	Improved chemo response in ovarian cancer; reduced dysbiosis	Modulated gut microbiota; enhanced drug response	Bi et al. (119)
*L. plantarum* (vaginal isolate)	*In vitro* (HeLa)	Suppressed proliferation and induced apoptosis	Induction of mitochondrial apoptosis pathway	Nami et al., 2014b (120)
*Lacticaseibacillus casei* LH23	*In vitro* (HeLa cells)	Suppressed proliferation; induced apoptosis	Mitochondrial apoptotic gene activation	Hu et al. (93)
*L. reuteri*	Oral (≥5.4 billion CFU, discontinued at HPV clearance)	No effect on HR-HPV clearance; may reduce abnormal smear rates	Possible immune modulation, but mechanism unclear	Ou et al. (121)
*B. adolescentis* SPM1005-A	*In vitro*	Anticancer activity	Induced apoptosis via modulation of pro-apoptotic genes	Cha et al. (122)
*L. casei* SR1, *L. casei* SR2, *L. paracasei* SR4	*In vitro* (HeLa cells); human milk isolates	Suppressed cervical cancer cell proliferation	Induction of apoptosis via expression of mitochondrial apoptotic genes	Dellino et al. (123)
*L. plantarum* 5BL	*In vitro* (HeLa); vaginal secretion isolate	Antiproliferative and pro-apoptotic activity	Activation of mitochondrial apoptosis pathways	Dellino et al. (123)
*Enterococcus faecalis* por1	*In vitro*; porcine intestinal isolate	Cytotoxic effects on gynecological cancer cells	Not fully characterized; likely via microbiome-mediated immune or apoptosis routes	Dellino et al. (123)

The microbiome-targeted therapies such as vaginal microbiota transplantation (VMT) and fecal microbiota transplantation (FMT) are gaining significant attention for their potential to enhance the efficacy of cancer immunotherapy and chemotherapy. Preclinical research and early phase clinical studies are beginning to unravel the modulatory roles of the microbiome in achieving better treatment response and reduction of side effects, in especially gynecological cancers and gastrointestinal cancers. The vagina’s microbial community consisting of the majority of species Lactobacillus plays a critical role in the ecosystem health of the vaginal tract and modulates risk of gynecological cancer such as ovarian and cervical cancer. Recent studies underpin the presence of major differences in the microbiota of the vagina and intratumoral compartment between cancer patients and healthy controls (110). Studies have also shown that ovarian cancer patients have a lower prevalence of the vaginal community dominated by Lactobacillus compared to the prevalence in cancer-free women, indicating that there may be a relationship between the development of cancer and vaginal dysbiosis. For example, high-throughput sequencing of tissue samples was used to identify a more diverse and rich microbiome in epithelial ovarian cancer (EOC) as compared to noncancerous tumors with specific bacteria the like Propionibacterium acnes implicated in tumor development (111). A study that included 187 participants revealed unique bacterial species that are more common in the fallopian tubes and on the ovarian surface of ovarian cancer patients, suggesting that alterations in the composition of microbes may be a source of carcinogenesis (111). VMT focuses on restoring a healthy microbial community in the vagina through the transplant of Lactobacillus communities from healthy donors. A recent randomized controlled trial has shown that donor microbiota engraftment in some women was associated with anti-inflammatory change in the gene expression of the vagina on exposure to VMT without antibiotics (124). This indicates the potential of VMT in the short-term treatment of vaginal dysbiosis, and possibly in reducing the risk of cancer by restoring homeostasis of the immune system.

Beyond VMT, Fecal Microbiota Transplantation (FMT) in cancer therapy involves transferring gut microbiota from healthy donors to patients to restore microbial diversity and function (125). It has shown promise in enhancing responses to immune checkpoint inhibitors (ICIs) and chemotherapy in various cancers (125). Although there is an increasing number of studies establishing the effectiveness of FMT in gastrointestinal cancers, melanoma, renal, colorectal, and genitourinary cancers (126), the impact of FMT interventions on gynecological cancer remains a gray area.

Lactobacillus-based probiotics is dominating research on microbiome-targeted therapeutic interventions for HPV clearance, vaginal microbiome restoration, cervical dysplasia, endometrial lesions and chemo/radiotherapy adjuncts (**Table 1**) (127). Verhoeven et al. (102) reported that oral administration of Lactobacillus casei Shirota contributed to the resolution of low-grade cervical lesions with associated cytological abnormalities in HPV-positive women. Indicating potential benefits, the HPV clearance occurred in 19% of controls versus 29% of probiotic users. In addition, a study including 100 women of reproductive age showed a significant decrease in HPV load, ameliorated vaginal inflammation, and improved clearance without observable side effects following vaginal *L. crispatus chen-01* transplantation (103). Similarly, Gao et al. (112) reported suppressed growth of HPV-positive human cervical cancer cells by Lactobacillus gasseri LGV03 isolated from the cervico-vagina of HPV-cleared women by modulates epithelial innate immune responses. Chronic exposure to Lactobacillus rhamnosus BMX-54 led to a significant increase in the resolution rate of cytological abnormalities associated with HPV as compared to short-term use and thus suggests the positive effect of long-term use of the probiotic in resolving abnormal cervical cell changes associated with HPV (113). In addition, the Lactobacillus rhamnosus BPL005 strain has been shown protective against endometrial infections with associated decreased PH as well as production of organic lactic acid which inhibits the growth of pathogenic bacteria (114). Laboratory investigation have demonstrated that the Supernatants of L. jensenii cultures decreased the viability of cervical cancer cell due to their inhibition of the growth of the cancer cells and apoptotic cell death, reduction of expression of the MMP9 gene and HPV oncogenes (115). In the same vein, the vaginal isolate of Lactococcus lactis probiotic candidate plays modulatory roles in ovarian cancer development L. lactis probiotic candidate downregulates TLR-4, miR-21 and miR-200b expression levels and shows correlation with the induction of apoptosis in the CAOV-4 cells (116).

The recombinant Lactococcus lactis expressing the HPV-16 E7 protein when administered by induced cellular and humoral immunity following intranasal administration thus providing protection against HPV-associated cervical cancer (117). Further, Kenneth et al. (128) showed that probiotics restored the gut microbiota by probiotic supplementation after antibiotic treatment improved the responsiveness to chemotherapy treatments and minimized chemoresistance, which offers a clue of direct influence of the microbial balance on the efficacy of drugs. Given this, certain strains such as Lactobacillus casei and L. paracasei have shown direct anticancer effects in models of cervical cancer through cell proliferation inhibition and apoptosis induction (118). Specifically, Bi et al. (119) reported that they were able to find evidence that Lactobacillus delbrueckii subsp. lactis added to treatment improved treatment outcomes through modulation of the gut environment, which, in turn, influenced tumor response to treatment. In the clinic, this probiotic strain has also been found to improve the radiotherapy-induced dysbiosis and gastrointestinal symptoms in women undergoing treatment for gynecological cancers, thus displaying a combined effect in promoting the effectiveness of treatment and reducing the unfavorable consequences of radiotherapy (119).

The wider applicability of microbiome modulation to human health is also highlighted by the results of work developing outside of the gynecologic discipline. Ambrose et al. (2025), for example, have shown that probiotics can improve wound healing and tissue regeneration including immune regulation, optimal epithelium healing and restoration of microbial balance. Although their study is focused on the dermatologic injury, the principles at play (targeted manipulation of microbiome, crosstalk between host and microbes, and personalized manipulation of immune responses) are directly applicable to the gynecologic oncology setting. These findings highlight that microbiome-directed interventions are part of a wider translational framework and support the rationale for integrating personalized microbial therapies into cancer prevention and treatment strategies.

Personalized microbiome interventions are emerging as an extension of precision therapeutics, enabling microbial modulation tailored to an individual’s microbial composition, immune profile, and treatment response (129). Rather than a generalized probiotic approach, these strategies involve targeted restoration of beneficial taxa, engineered microbial consortia, or selective metabolic modulation guided by microbiome profiling. Such personalized microbial therapeutics complement existing cancer prevention and treatment pathways, offering the possibility of enhancing therapy effectiveness, improving immune responsiveness, and reducing treatment-associated toxicity (130).

### 6.1 Microbiome engineering and targeted intervention in gynecological oncology

Recent literature has suggested the promise of microbiome engineering in tumor-associated microbial communities in gynecological cancers. Targeted delivery of engineered vaginal Lactobacillus strains, and antimicrobial molecules including antiviral peptides and cytokine-modulating compounds form a therapeutic delivery system for reducing the persistence of HPV and boosting mucosal immunity (131, 132). Evidence has demonstrated the potential of reversing dysbiosis by restoring the depletion of Lactobacillus species and decreasing the pro-inflammatory taxa associated with endometrial and cervical carcinogenesis following exposure to synthetic microbial consortia (133). According to Wu et al. (134) CRISPR-based systems enabled targeted inhibition of tumor-promoting metabolites such as lipopolysaccharide-induced inflammatory mediators and sialidases. Beyond local interventions, engineered bacteria have the capability of producing immunoregulatory molecules and enhances chemotherapeutic sensitivity as an adjuvant therapy for ovarian cancer (135). Though these approaches are largely preclinical in design, they can be integrated with existing screening, HPV treatment and immunotherapy pathways. These will require further clinical trials and investigations, regulatory oversight for genetically modified vaginal strains, and standardized safety evaluation (136).

### 6.2 Limitations and future possibilities in therapeutic microbiome engineering

Microbiome engineering, which involves manipulating microbial communities or their functions to treat disease holds immense therapeutic promise across many medical fields. However, despite rapid scientific advances, significant limitations and challenges remain before these therapies can be widely and safely implemented. The study of the microbiome and sequencing protocols have revealed the associated complexity and variability that pose several limitations and methodological problems. This complexity complicates the identification of specific disease-causing microbial signature, and beneficial strain as a therapeutic target. Daily fluctuations and sampling challenges across different gut regions further obscure accurate characterization (137). Variation in sample collection is also one of the most methodological challenges of microbiome studies of gynecological cancers. Depending on the anatomical site of sampling (vaginal swab, cervical brush, endometrial biopsy, etc.), time, and handling, the microbial populations found can differ significantly.

The sequencing technology and the choice of bioinformatics pipeline also influence the outcomes. Although metagenomics provides more comprehensive taxonomic and functional understanding, it also presents a number of difficulties, such as the need for efficient taxonomic annotation databases and efficient host DNA extraction. 16S rRNA gene sequencing is popular and provides good agreement with quantitative PCR for important taxa, especially the V1–V3 or V3–V4 regions. Importantly, many studies lack sufficient details on their molecular and bioinformatic methods, which presents challenges for reproducibility and cross-study comparisons (138). It is consequently crucial that protocols be standardized and publicly reported in order for the field to advance and enable the meaningful synthesis of findings across investigations. Therefore, this decade must see an improvement in analytical tools and computational platforms. The development of standardized, high-resolution sequencing methods and robust bioinformatic pipelines will improve reproducibility and functional interpretation of microbiome data. Machine learning and systems biology approaches can help decipher complex microbial interactions and predict therapeutic responses, guiding rational design of microbiome interventions (139; 140).

Further, microbiome-based therapies such as fecal microbiota transplantation (FMT) carry risks of transmitting undetected or emerging pathogens (137). Long-term ecological stability of introduced microbes is uncertain, raising concerns about sustained efficacy and unintended consequences on native microbial communities (141). Engineered bacteria, while promising, require rigorous safety evaluation to avoid off-target effects or horizontal gene transfer. Regulatory frameworks for such living therapeutics remain underdeveloped, delaying clinical translation (142). This calls for deliberate increased attention on safety by researchers and policy makers alike.

There is a lack of standardization in microbial product composition, dosing, and delivery methods. For example, up to 50% of microbial sequences in human stool cannot be mapped, and the functional roles of many microbes remain unknown. Manufacturing microbial consortia that mimic natural, stable communities is complex, and scaling production while maintaining quality is challenging. Additionally, microbial metabolites often have poor stability, unpleasant taste or odor, and difficulties with targeted delivery and dosing (142). Moving beyond single-strain probiotics, microbial consortia offer the potential to restore diverse and stable microbiomes resembling natural ecosystems (137). Engineered bacteria using advanced tools like CRISPR-Cas can be programmed to produce therapeutic molecules, sense disease markers, or modulate host immunity with precision. For example, *E. coli* strains have been engineered to detect gut inflammation and respond by releasing immunomodulatory compounds (142). Beyond gynecological diseases, microbiome engineering holds promise for metabolic disorders, neurodegenerative diseases, dermatology, and infectious diseases; therefore, multifunctional microbial therapies that combine sensing, therapeutic delivery, and real-time monitoring represent an exciting frontier (137, 141**;** 140).

In addition, high-throughput sequencing and metagenomic analyses are powerful tools but have inherent limitations. Targeting only select regions of the 16S rRNA gene can miss key species or genetic variations, and different sequencing platforms and bioinformatic pipelines produce variable results, complicating reproducibility and interpretation (143). The large volume of data demands high-performance computing and specialized expertise, which are not universally accessible (139).

The variations in individual responses is also a noteworthy challenge. Therapeutic responses vary widely among individuals due to differences in microbiome composition and host factors (140). Besides, individual microbiomes are highly heterogeneous, and factors such as age, hormones, gender, genetics, diet, environment, and lifestyle defeat the development of “one-size-fits-all” intervention. Such heterogeneity also complicates the determination of microbial signatures of disease and large, well-characterized cohorts are not easily distinguishable between variation and disease-related changes. One such case was a study of vaginal microbiota in gynecological cancer analysis, where, although some taxa (Firmicutes and *Lactobacillus* were enriched, and others (Bacteroidetes, Proteobacteria and *Prevotella*) were depleted in the cancer patients, the menopause status did not modulate these patterns suggesting that disease status may be more important than some host factors (101).

Building on the framework proposed by Jurja et al. (144), who combined subjective questionnaires with objective biomarker assessments and longitudinal follow-up, a similar multidimensional approach is particularly relevant in gynecologic microbiome research. Their model underscores the importance of capturing patient-level variability, which is evident in microbiome–cancer interactions. For instance, pre- and post-menopausal women differ substantially in estrogen levels and vaginal pH, influencing the stability of Lactobacillus-dominated communities and susceptibility to dysbiosis. Tumor subtypes also show contrasting microbial fingerprints: high-grade serous ovarian cancers commonly exhibit enrichment of Proteobacteria and inflammatory anaerobes, whereas endometrial cancers often correlate with gut-mediated estrogen metabolism. HPV-positive patients tend to show microbiomes characterized by Lactobacillus depletion and anaerobe overgrowth, while HPV-independent vulvar or endometrial cancers follow divergent dysbiotic pathways. Geographical and ethnic differences further shape baseline vaginal community state types, diet, and gut estrobolome composition, producing distinct microbial risk profiles across populations. These contrasts reinforce Jurja et al. (144)’s argument that integrating patient-reported data with objective molecular testing and extending this through longitudinal sampling would allow more accurate stratification and prediction of microbiome intervention effectiveness across diverse gynecologic cancer subgroups.

On the other hand, the cross-sectional or observational design of most existing microbiome studies in gynecological oncology limits the ability to draw causality conclusions. This gap has since started to close with the introduction of genetic techniques to obtain inferences of causality, e.g. Mendelian randomization (MR). As one recent MR study established, there are 33 potential causative relationships between gut microbiota and gynecological malignancies, with some of these genera being protective and others risk factors. Adding to the complexity of understanding causality, the research also found some bidirectional effect, whereby, the existence of cancer may alter the composition of the gut microbiota (145).

The molecular processes that connect the microbiome to carcinogenesis need to be clarified immediately through mechanistic research employing integrated multi-omics techniques, animal tests, and *in vitro* models. According to experimental research, for example, some bacteria have the ability to change metabolic pathways in the reproductive tract and produce pro-inflammatory cytokines, which may encourage the development of cancer. The combination of HPV infection, local inflammation, and microbiome composition can drive cervical carcinogenesis, according to multi-omics techniques. That is why functional investigations are needed to complement observational data (35). Repeated sampling and longitudinal studies are recommended to enhance the identification of biomarkers. Moreover, integration of multi-omics data (metabolomics, genomes) can help to clarify the functional implication of the identified changes in microbes, and their relation with the pathophysiology of cancer (35). The implementation of personalized approaches has also become an imperative despite the pre-intervention microbiome profiling and the personalized therapies, which raises the complexity and the cost (137). Future therapies will probably involve detailed pre-treatment profiling of the microbiome to individual microbe landscapes to better personalize therapeutic interventions to reduce side effects and increase efficacy. Breakthroughs in smart capsules technology and the development of better sampling techniques will enable microbiomes to be characterized more accurately and completely. Integration with dietary and lifestyle changes could promote better treatment outcomes and sustainability. Since they must know the evolving knowledge on microbiome functioning, potential risks, and the uncertain long-term consequences of altering microbial communities, in this case, informed consent is challenging. The potential that microbiome data could be linked to genetic and clinical data raises privacy issues as it brings into question the security and confidentiality of data. Commercialization of products based on microbiomes, data ownership, and biobank governance are all areas that would need proper regulatory standards and continued ethical review. The insufficient information on the safety and efficacy of microbiome-based treatment of cancer patients over a long period of time makes the risk-benefit analysis particularly difficult. To ensure that health disparities do not increase, it is imperative to ensure equal selection of participants and access to the said therapies. Ethical issues centering on informed consent, donor screening for FMT, and public acceptance have cannot be neglected-–- especially concerning genetically-modified organisms (GMOs)-–and must inform future studies and practice (137, 142). Establishing clear regulatory guidelines and safety standards for microbiome-based therapeutics, including engineered microbes, is critical for clinical adoption. Ongoing clinical trials and real-world evidence will inform risk-benefit assessments and best practices for donor screening, manufacturing, and long-term monitoring (141, 142).

## 7 Conclusion

The evidence presented in this review shows that the human microbiome, especially the vaginal, endometrial, ovarian, and gut microbiome, play a central role in the development and advancement of gynecological malignancies. The loss of protective microbial species and increased representation of anaerobic or pathogenic taxa is a common feature of dysbiosis, which has been identified in cervical, endometrial, and ovarian cancers. Mechanistically, such microbial changes induce carcinogenesis through multiple related pathways: chronic inflammation, alteration of epithelial barrier integrity, regulation of local and systemic immunity, and interference with hormone metabolism, particularly estrogen cycling. The pro-inflammatory signaling cascades and DNA damage is perpetuated by activation of pattern-recognition receptors (Toll-like receptors) by microbial products, and microbial metabolites and byproducts may also affect cell proliferation, apoptosis, and genomic stability. The therapeutic implication of this relationship is anchored on the potential of microbiome as a potential adjunct and possible intervention in the treatment of gynecological cancers. Microbial signatures can be used as non-invasive biomarkers of early detection, risk stratification, and to monitor responses to therapy. Their clinical usefulness will, however, hinge on scientific validations in various, longitudinal populations and how they are standardized in terms of the sampling and analytical protocols. The potential interventions that could be used to restore microbial homeostasis and alter cancer risk or progression are microbiome-based interventions, such as probiotics, prebiotics, dietary modification, and in some instances microbiota transplantation. However, these are still underdeveloped in gynecological oncology research and practice. In essence, integrating microbial ecology into gynecologic cancer research and care offers a new precision-based framework capable of improving prevention, refining diagnosis, enhancing therapeutic response, and ultimately transforming patient outcomes.
